# Comparing object lifting kinematics and the size–weight illusion between physical reality and virtual reality

**DOI:** 10.3758/s13414-025-03091-w

**Published:** 2025-05-27

**Authors:** David John Harris, Callum Aaron O’Malley, Tom Arthur, Jack Evans, Gavin Buckingham

**Affiliations:** School of Public Health and Sport Sciences, Faculty of Health and Life Sciences, Medical School, University of Exeter, St Luke’s Campus, Exeter, EX1 2LU UK

**Keywords:** Perception, Action, XR, VR, Extended reality, Motor

## Abstract

This study compared the size–weight illusion (SWI) and object lifting kinematics between physical and virtual conditions, shedding light on the nuanced disparities in perception and action across different environmental mediums. We examined whether prior expectations about object weight based on size cues, which affect the experience of real-world object interactions, are different in virtual reality (VR). Employing a highly realistic virtual environment with precisely matched visual size and haptic cues, we tested the hypothesis that VR, which may be experienced as uncertain, unfamiliar, or unpredictable, would induce a smaller SWI due to a diminished effect of prior expectations. Participants (*N* = 25) reported the felt heaviness of lifted objects that varied in both volume and mass in physical reality and a VR environment. Reach and lift kinematics, and self-reported presence, were also recorded. Our findings showed no differences between how participants perceived the SWI between real and virtual environments, although there was a trend towards a smaller illusion in VR. Contrary to our predictions, participants who experienced more presence in VR did not experience a larger SWI—instead, the inverse relationship was observed. Notably, differences in reach velocities between physical and virtual conditions suggested a more controlled approach in VR. These findings highlight the intricate relationship between immersion and sensorimotor processes in virtual environments, emphasising the need for deeper exploration into the underlying mechanisms that shape human interactions with immersive technologies, particularly the prior expectations associated with virtual environments.

## Introduction

Extended reality (XR) environments have gained popularity as a versatile training method across industries like surgery (Alaker et al., 2016), aviation (Harris et al., 2023), sport (Neumann et al., 2018), and related fields. XR technologies are also now widespread in psychological research, promising more ecologically valid, yet still tightly controlled, experimental tasks (Buckingham, 2019; Mangalam et al., 2023; Naylor et al., 2022; Snow & Culham, 2021). Our understanding of how these technologies impact perception and action is, however, still in its infancy. In particular, we do not yet know how the brain interprets computer-generated sensory information and how our perception of virtual worlds might differ from that of physical reality (Giesel et al., 2020; Harris et al., 2019; Mangalam et al., 2021). Perception and action are known to be strongly influenced by expectations (‘priors’) formed through experiential learning (Buckingham, 2014; de Lange et al., 2018; Hill & Johnston, 2007; Wolpert, 2007). Indeed, Mary Peterson’s work has demonstrated how top-down processes, such as prior knowledge and expectations, can shape perceptual organization, including figure–ground segregation and object recognition (Peterson, 1999; Peterson & Gibson, 1994). These findings suggest that our brains rely on learned patterns and contextual cues to resolve perceptual ambiguity, a mechanism that may operate differently in XR environments due to the novel and often less predictable nature of computer-generated sensory input. Therefore, it is unknown whether individuals hold similar priors when in XR environments, where the world appears real but we know that it is not. Even subtle variations in the way priors guide perception in XR could have profound downstream effects, potentially disrupting its expanding applications in motor skill training, rehabilitation, and psychological experimentation. The present study examined whether the prior expectations that guide perceptions of weight during the size–weight illusion are altered in XR.

The term ‘XR’ encompasses diverse technologies that alter the human experience by blending the physical and digital worlds. Within the XR spectrum, mixed reality (MR), augmented reality (AR), and virtual reality (VR) represent distinct but interconnected concepts (Milgram & Kishino, 1994; Slater & Wilbur, 1997). For instance, MR integrates virtual and real-world elements, whereas AR overlays digital information onto the real environment. Meanwhile, VR immerses users entirely in a simulated environment, isolating them from the physical world. One issue is that compelling XR experiences depend upon deliberately misleading our sensory systems to create illusory perceptions of motion or three-dimensional space (Harris et al., 2019). For instance, haptic feedback is derived from the active experience of touch, but VR peripherals can only approximate haptic information. Similar issues are present for vision, where conflicting display cues relating to depth and disrupted eye–lens coordination can distort elements of perception (Eadie et al., 2000; Wann et al., 1995). As such, XR technologies can recreate sensory inputs sufficiently well so as to create a compelling experience, as our brain ‘fills in the gaps’, but even subtle uncertainties and mismatches between real and virtual inputs will inevitably alter our actions (Giesel et al., 2020). Preliminary evidence has suggested that the altered perceptual environment of VR does indeed affect our motor behaviours (Giesel et al., 2020; Kober et al., 2022). An emerging pattern appears to be that movements in VR may be more exaggerated and less fluent (Brock et al., 2023; Harris et al., 2020; Magdalon et al., 2011; Weir et al., 1991), which could indicate a fundamentally different mode of action control. A proposed explanation is that movement control in VR uses information derived from the ventral visual stream, as opposed to the dorsal ‘vision-for-action’ stream, making it more consciously controlled and less automatic (Beck et al., 2010; Brock et al., 2023; Harris et al., 2019).

A second, potentially more pervasive, issue is how priors shape our perception in XR, and whether this differs from the real world. The critical role of internal predictive models in shaping sensorimotor functions and processing sensory input has been well-established in the literature (Clark, 2013; Friston, 2010; Körding & Wolpert, 2004). Humans use predictive models of themselves and their environment to quickly and robustly make sense of incoming sensory information (de Lange et al., 2018). As a result, our perception of the world is a joint estimate of bottom-up sensations and top-down expectations (Clark, 2013; Friston, 2005; Helmholtz, 1860; Seth, 2015). Unusual perceptual phenomena like the McGurk effect (McGurk & MacDonald, 1976) or hollow-face illusion (Gregory, 1970) are often cited as examples of prior expectations innervating perceptual experience. Notably, the ‘size–weight illusion’ (SWI) has been extensively studied over decades, revealing that acquired knowledge about the perceived weight of larger versus smaller objects significantly influences both fingertip forces exerted during object lifting and the corresponding perceptual experience of weight (Buckingham, 2014; Ellis & Lederman, 1999; Flanagan & Beltzner, 2000). When objects with differing sizes but equivalent weights are lifted, this error in prediction leads to the experience of smaller objects feeling heavier than similarly weighted larger objects (Charpentier, 1891). Priors operate within a probabilistic framework, wherein more precise beliefs exert a stronger influence on perception, while less precise beliefs are more susceptible to override (Knill & Pouget, 2004; Yu & Dayan, 2005). Priors exhibit malleability and context specificity, rendering them highly responsive to the surrounding environment and our knowledge/expectations about it (Trapp & Bar, 2015). Consequently, a belief indicating novelty, unfamiliarity, or unpredictability in the current context can propagate cascading effects on the delicate balance between top-down predictions and bottom-up sensations (Behrens et al., 2007; Yon & Frith, 2021). Herein lies the concern for XR. For instance, the distinction between perceiving virtual objects as real things or images, and how this affects our expectations of their physical properties, is a concern (Snow & Culham, 2021). In addition, virtual worlds are, often overtly, not beholden to the laws of the physical environment, which may further affect the way people make predictions about sensory input, causal regularities in the world, and their own action capabilities (Yarossi et al., 2021). Together, these factors suggest that immersion in a virtual world may influence the relative balance between top-down and bottom-up influences on perception.

### The present study

In the present work we sought to explore how priors shape perception in XR, by comparing the SWI in physical reality with high-fidelity VR. The SWI paradigm is ideally suited to capture the extent to which priors shape perception, because the magnitude of the illusion is directly linked to our prior expectations. Consequently, if prior expectations about the weight/density of objects in VR are relatively uncertain (compared with sensory inputs) a *smaller* SWI would be expected, compared to physical reality. By contrast, if individuals are generally uncertain about sensory inputs in VR and rely more on prior expectations (e.g., see Rzepka et al., 2022), a *larger* SWI effect would be expected in VR.

Several previous studies have used VR as an experimental tool to examine the origins of weight illusions (Buckingham, 2019; Naylor et al., 2022; Rohrbach et al., 2021; van Polanen & Davare, 2019). This work has exploited the potential of VR for controlling visual cues to create mismatches between, for example, visual and tactile cues to object material (Naylor et al., 2022) or visual and haptic cues to object volume (Buckingham, 2019). In a similar vein, Rohrbach et al. (2021) showed that equally sized AR holographic cubes overlaid onto physical cubes initially eliminated the SWI when lifting differently sized, but equally weighted, real cubes. These studies have shown that virtual stimuli still induce weight illusions, indicating that our priors about object mass and density do, to some degree at least, hold in VR. None of the studies have, however, directly compared the magnitude of perceptual illusions between virtual and physical reality.

The only existing study to make a direct comparison, by Heineken and Schulte (2007), indicated that the magnitude of the SWI may be affected by presentation condition when comparing physical reality, desktop ‘VR’ (flat screen), and head-mounted VR. The authors proposed that the size of the illusion depended on the perceived ‘realism’ of the visual presentation, with larger effects of object size on perceived weight in natural reality and the more immersive virtual conditions. In this study, conducted with an older VR system, participants interacted with objects by employing a ‘hooking stick’ rather than natural grasping. To delve deeper into this matter, we utilised a higher fidelity VR environment that facilitated more natural interactions. Additionally, we gathered presence ratings to investigate whether perceived ‘realism’ played a critical role in determining the SWI, as suggested by Heineken and Schulte (2007). Moreover, we examined movement kinematics to assess whether the previously observed trend of slower and more controlled movements in XR held true for this specific task.

Hypothesis

- **H**_**1**_—Following the findings of by Heineken and Schulte (2007), we predict that the magnitude of the SWI will be *smaller* in the VR condition because of relatively weaker expectations about the physical properties (e.g., heaviness, density) of objects.
- **H**_**2**_—Due to sensory uncertainty (e.g., hand visualisation and impaired depth cues) in VR, lifting kinematics will be slower and more controlled (lower maximum lift velocity and maximum reach velocity as well as longer time to maximum lift and reach velocities).
- **H**_**3**_—Higher self-reported presence scores will correlate with a larger SWI in the VR condition, because expectations will be stronger when participants feel more immersed in the environment.

## Methods

### Transparency and openness

We report sample-size determination, data exclusions, and all manipulations in the study. All data and analysis code are available online (https://osf.io/8gty7/). The main hypotheses were preregistered prior to data collection and the preregistration document is available online (https://osf.io/7jyxk). Data were analysed using RStudio (Version 1.4.1106; R Core Team, 2017).

### Design

We used a repeated measures design in which all participants took part in both conditions, the order of which was counterbalanced across participants. The conditions were object lifting in physical reality (‘*real’*) and in a virtual environment (‘*virtual’*). As the virtual environment involved grasping co-located physical objects, it could be strictly termed as a mixed reality environment. But as the visual environment was entirely virtual and for consistency with the relevant prior literature, we refer to this condition as ‘VR’ throughout.

### Participants

We employed an opportunity sample of individuals, recruited from the student population at the host University. Twenty-five participants (15 women) took part in the experiment. Participants had a mean age of 20.8 years (*SD* = 2.4, range: 18–30). Of the 25 participants, 24 were right-handed and 18 reported having used VR before. Participants were provided with details of the study and gave written informed consent on the day of the testing visit, which lasted around 45 min. They were compensated £20 for their time. Ethical approval was obtained from the departmental Ethics Committee prior to data collection (REF: 3,474,669) which took place between January and March 2024.

The sample size was chosen based on an a priori power calculation using the software G*Power (Faul et al., 2007). The effect size of interest for the power calculation was based on previous studies of the size–weight illusion and the magnitude of weight-estimation errors. SWI effects are typically large, with a meta-analysis by Saccone et al. (2019) reporting a pooled effect of *d* = 1.82. In the most similar previous study to the present work, Heineken and Schulte (2007) reported a very large main effect (η_p_^2^ = 0.57) when comparing the magnitude of the size–weight illusion across different visual presentation mediums. As a conservative adjustment, we selected an effect size conventionally considered medium to large (*d*_*z*_ = 0.7) but which was still considerably smaller than either of these effects, giving us additional power to also detect more moderate effects. To achieve 90% power in a paired *t* test, given *d*_z_ = 0.7 and α = 0.05, 24 participants were required.

### Materials

#### Virtual reality size–weight illusion stimuli

Adhering to the procedures described in Arthur et al. (2020) and Allen et al. (2023), participants were asked to lift and judge the weight of four tall black plastic cylinders. Objects were of identical height (7.5 cm) but differed in their physical diameter (small: 5 cm, large: 10 cm) and mass (light: 355 g, heavy: 490 g), creating a total of four ‘test’ items (i.e., small and heavy, small and light, large and heavy, large and light). Crucially, these test objects were used in both study conditions (i.e., participants lifted exactly the same physical items in the VR and real-world trial environments). This meant that the only differences between the two experimental conditions was in the visual presentation, and therefore any disparity in behaviours could not be attributed to haptic differences. The objects were filled with packing foam and lead shot, with the centre of mass balanced around the centre of the object. Five practice/washout trials were conducted at the start of each of the four blocks of trials using an intermediate-sized 490-g black plastic cylinder (7.5 cm tall and 7.5 cm in diameter).

**Fig. 1:**
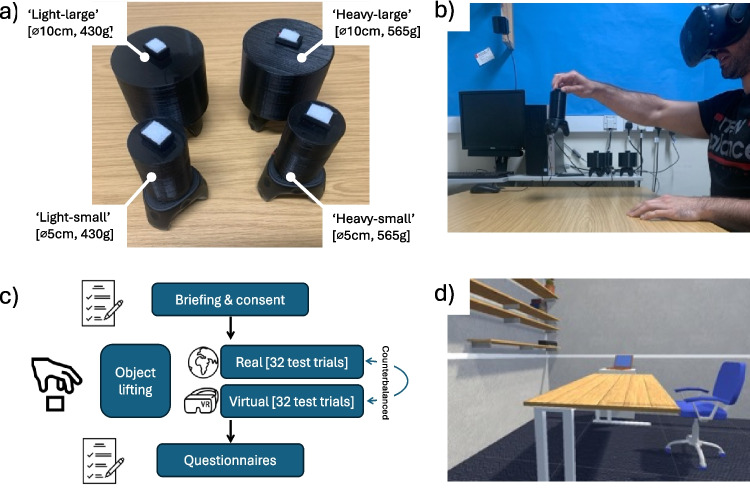
Experimental stimuli and design. *Note*: Panel **a)** shows the experimental stimuli of two sizes and weights. Panel **b)** shows a participant completing the lifting task. Panel **c)** illustrates the experimental design. Panel **d)** is a view inside the virtual environment, which was closely matched to the real lab

To create the virtual objects, an HTC Vive tracking device (HTC Corporation, Taiwan) was attached to the base of each real object (see Fig. 1a) which allowed its position and rotation to be measured and recreated exactly in virtual reality. The virtual objects were exactly matched in size with the actual physical objects, creating a ‘mixed-reality’ task in which the participant was simultaneously lifting virtual and physical objects. The dimensions of the Vive 3.0 trackers were 70.9 × 79.0 × 44.1 mm, and they weigh 75 g, taking the total weight of the light and heavy objects to 430 g and 565 g, respectively. Independent testing has supported the accuracy of the trackers for accurate visualization, even during more vigorous activities (Merker et al., 2023). The tracker was screwed to the base of the objects in the VR and real conditions.

During the VR condition, participants viewed the digital recreations via a VR head-mounted-display (HMD) in a bespoke immersive VR game environment, which was designed to look like a duplicate of the testing laboratory. The VR task was developed using the Unity 2019.2.12 gaming engine (Unity Technologies, CA) and C#. We employed an HTC Vive Pro Eye headset (HTC Corporation, Taiwan), a high-precision virtual reality (VR) system known for its effectiveness in tasks involving small-area movement research (Niehorster et al., 2017). The Pro Eye headset is consumer-grade, 6-degrees-of-freedom system that immerses users in a 360° environment with a 110° field of view. Graphics were generated using an HP EliteDesk PC running Windows 10, equipped with an Intel i7 processor and a Titan V graphics card (NVIDIA Corp., Santa Clara, CA). Headset and Vive tracker positions and rotations were monitored at 90 Hz via the three ‘lighthouse’ base stations. The Unity environment and videos of the task are accessible online at (https://osf.io/8gty7/).

### Measures

#### Perceived heaviness

##### Heaviness ratings

After each lift, participants provided a verbal numerical judgment of the perceived heaviness of the object using a numerical rating on a scale of their own choosing (i.e., *absolute magnitude estimation*; Zwislocki & Goodman, 1980). Participants were advised that they had the flexibility to use any numbers but were instructed to maintain a uniform rating scale across conditions, and that larger numbers represented heavier weights. This methodology aligns with the approach of numerous weight illusion studies, allowing the capture of subjective judgments while facilitating a standardized, quantifiable measure that minimizes ratio scaling biases (Buckingham, 2019; Buckingham et al., 2011). This measure can also be standardized across conditions through the use of *z* scores.

##### SWI score

For our primary dependent variable, a SWI score was calculated from the raw heaviness ratings to create an index that represented the magnitude of the illusion. Following the procedure outlined in Hassan et al. (2020), the SWI score was the number of grams weight difference perceived per cubic cm of volume change. We regressed the raw heaviness ratings onto object weight (in grams) and object volume (in cm^3^). We subsequently extracted the beta (slope) coefficient for each relationship (per participant, per condition). We then computed:

$$SWI-score=-\left(1/bW*bV\right),$$

where *bW* is the beta for Weight and *bV* is the beta for Volume. The sign is reversed so that a larger score represents a larger weight illusion. The SWI score quantifies the strength of the illusion by assessing how much a change in an object’s size (volume) influences perceived heaviness, relative to its actual weight. A higher SWI score therefore indicates a stronger illusion. Compared with a simple difference score, this scaled measure more accurately detects changes in the size–weight illusion, allowing for comparison between individuals, whilst still accounting for individual differences in real weight perception.

#### Presence

A sense of presence refers to the subjective feeling of being physically present in the computer-generated environment (Slater, 2018). Higher levels of presence are linked to hardware and software that supports greater immersion, such as high visual realism and greater interactivity. The Slater–Usoh–Steed (SUS) Presence questionnaire (Slater et al., 1998; Usoh et al., 1999) was chosen as a measure of participants’ sense of presence in the virtual environment. The SUS contains six questions that relate to three themes: i) the sense of being in the virtual environment; ii) the extent to which the virtual environment becomes the dominant reality; and iii) the extent to which the virtual environment is remembered as a ‘place’. Questions are answered on a 1 to 7 scale, where the higher score indicates greater presence. The presence score is taken as the number of answers that have a score of 6 or 7.

#### Reach and lift kinematics

Reach and lift kinematics were recorded from the Vive trackers attached to the participant’s wrist and lifted object. As in Arthur et al. (2020), raw positional data from the tracker were smoothed using a dual-pass, zero-phase lag 10-Hz Butterworth filter. Hand and object velocity were then calculated by differentiating position with respect to time. Next, reach and lift movement phases were segmented for each trial. The reach phase was defined as beginning when hand velocity first exceeded 50 mm/s for three consecutive frames and concluded when velocity next fell below 50 mm/s for three consecutive frames. The lift phase was defined as the time point where both hand and object velocity first exceeded 50 mm/s until the time point at which the object reached its maximum vertical position. The maximum reach velocity (MRV) and maximum lift velocity (MLV) values were then recorded, as were the time points at which these events occurred (as a per cent of total movement time).

### Procedure

Participants attended the lab for a single visit lasting around 40 min. After having the experimental procedures explained, they provided written informed consent and demographic information. For the lifting trials, participants were seated at a table in a height-adjustable chair. They began each trial with their eyes closed and their preferred hand flat on the table surface. The experimenter placed an object in front of them and an auditory tone signalled to the participant to open their eyes and reach out to pick up the object. Participants were instructed to grasp the handle with just the thumb and index finger of their preferred hand and to lift the object several centimetres off the table in a smooth, controlled, and confident manner. Participants held the object for 2 s and then replaced it on the table. They then provided their numerical rating of heaviness, which was recorded by the experimenter. Prior to the first lift in each condition, participants were also asked to verbally estimate the expected weight of the objects (small, medium, large) to check that the stimuli induced the standard expectation that larger objects will be heavier. Plots of the results are included in Supplementary File 1, which confirmed that this was the case indicating that there was no systematic difference in expectations about weight prior to the lifts. Participants lifted the medium sized object five times (washout trials) followed by eight lifts of each of the four objects (i.e., 32 test trials) in one of four pseudo-randomized orders (see OSF). This procedure was repeated for the *real* and *virtual* conditions*.* After the virtual condition, participants also completed a simulator sickness questionnaire, to check whether they had experienced any adverse symptoms (a plot of these results is available in Supplementary File 3 on the OSF page).

### Data analysis

Data were checked for substantial deviations from normality prior to analyses. Outlying values (> 3.29 standard deviations from the mean) were winsorised using the DescTools package for R (Signorell et al., 2019) as outlined in Pek et al. (2018). Cohen’s *d* effect sizes are reported for all *t* tests and partial eta-squared (η_p_^2^) for all analyses of variance (ANOVAs). All statistical tests were conducted with alpha set at *p* < 0.05 and are reported alongside a Bayes factor computation, which illustrates the strength of evidence in favour of the alternative/null hypotheses. Bayesian analyses were conducted using the BayesFactor (BF) package (Morey & Rouder, 2015). A Cauchy prior (centred on zero, with a scale of 0.707) was used for paired *t* tests and Jeffreys–Zellner–Siow prior for ANOVAs. We report BF_10_ (i.e., evidence in favour of the alternative hypothesis). One participant had missing kinematic data for the real condition, so was excluded for the kinematic analyses only.

## Results

### Perceptual experience of heaviness

**Fig. 2:**
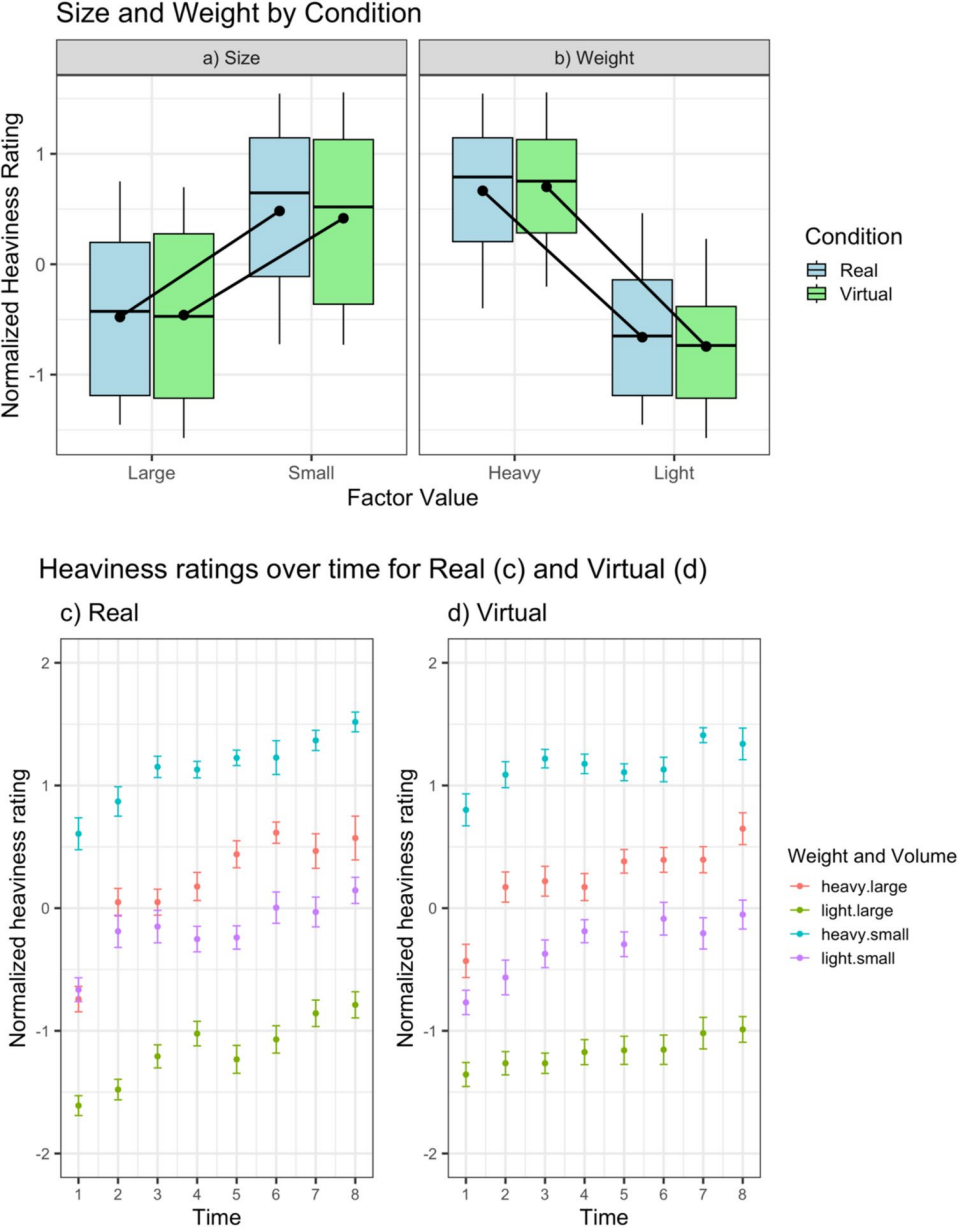
Heaviness ratings. *Note.* In panels **a)** and **b)**, boxes represent the interquartile range of the data and the median (thick black line), with the whiskers extending to the maximum/minimum values. The black points and connecting lines represent the mean values for each condition. In **c)** and **d)**, each point represents the mean rating at a specific time point, and the error bars indicate the standard error of the mean. (Colour figure online)

First we examined the effect of object size, mass, and lifting condition (real/virtual) on *z*-scored object heaviness ratings (see Fig. 2). There were significant main effects for both weight, *F*(1, 240) = 848.13, *p* < 0.001, η_p_^2^ = 0.78, BF_10_ = 6.90 × 10^35^, and size, *F*(2, 240) = 187.20, *p* < 0.001, η_p_^2^ = 0.61, BF_10_ = 4.73 × 10^13^, indicating that participants detected both the real and illusory differences in mass. There was no main effect of condition, *F*(1, 240) = 0.07, *p* = 0.79, η_p_^2^ < 0.01, BF_10_ = 0.14, and no interaction between condition and size, *F*(2, 240) = 1.76, *p* = 0.18, η_p_^2^ = 0.01, BF_10_ = 0.33, or condition and weight, *F*(1, 240) = 1.58, *p* = 0.21, η_p_^2^ = 0.01, BF_10_ = 0.89, indicating that virtual condition did not alter the experience of real or illusory mass. There was also no evidence of an interaction between size and weight, *F*(1, 240) = 0.05, *p* = 0.83, η_p_^2^ = 0.00, BF_10_ = 0.21, or between size, weight, and condition, *F*(1, 240) = 0.33, *p* = 0.57, η_p_^2^ = 0.00, BF_10_ = 0.23.

### *SWI score*^1^

**Fig. 3:**
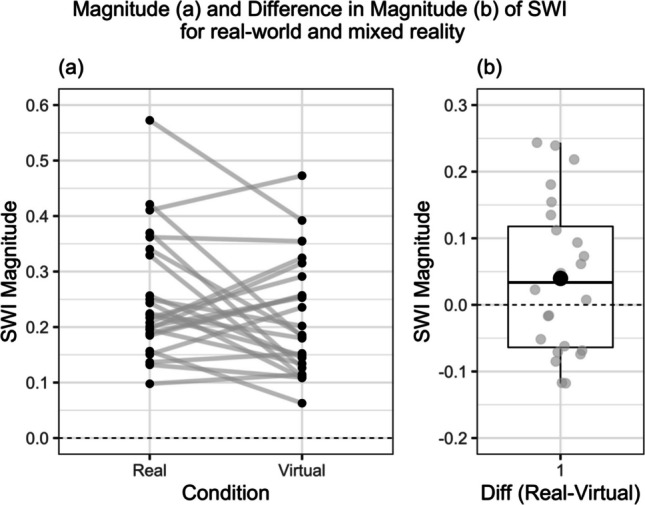
Comparison of SWI between conditions. *Note.* Panel **a)** shows raw data points. In panel **b)**, the box represents the interquartile range of the data and the median (thick black line) and mean (black dot), with the whiskers extending to the maximum/minimum values

To address our primary research question, we ran a paired *t* test to compare the SWI scores between real and virtual conditions, mean difference = 0.05; 95% CI(− 4.11e − 04, 0.11). The test indicated that there was a small reduction in SWI magnitude in VR (see Fig. 3), but this effect did not reach statistical significance, *t*(24) = 2.05, *p* = 0.052; *d*_z_ = 0.42. A Bayesian *t* test, using a Cauchy prior, indicated that there was only anecdotal evidence in favour of the alternative hypothesis (BF_10_ = 1.25).

### Presence

**Fig. 4:**
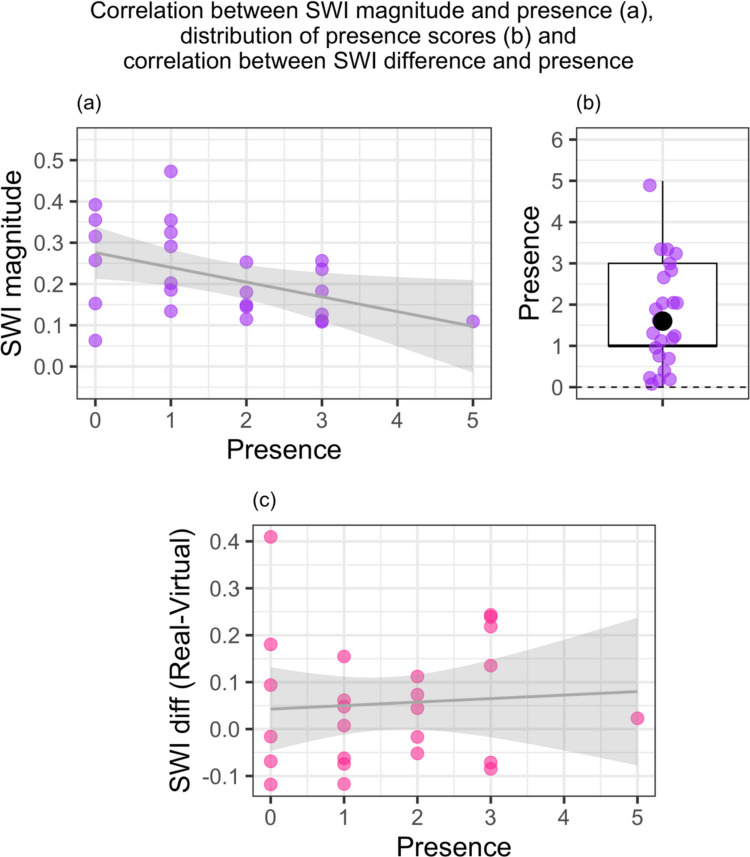
Relationship between SWI and presence. *Note.* Presence scores correspond to the number of categories in which participants provided a response of 6 or 7 (i.e., strongly felt the virtual environment was reality). In the distribution plot **(b)**, 0–6 represents the full range of possible mean values, with the box indicating the interquartile range of the data and the median (thick black line) and mean (black dot), with the whiskers extending to the maximum/minimum values

Next, we fitted a regression model to examine whether presence scores predicted the size of the SWI in the VR condition. The model explained a statistically significant and moderate proportion of variance, *F*(1, 23) = 5.76, *p* = 0.025, beta =  − 0.04, 95% CI(− 0.07, − 4.93e − 03), *R*^2^ = 0.20, BF_10_ = 2.66, indicating that at higher presence scores, the SWI magnitude was smaller (see Fig. 4a–b).

Next, we examined whether presence predicted the *difference* in the SWI magnitude between conditions (real minus virtual), to examine whether the sense of presence affected whether the SWI increased or decreased in VR (see Fig. 4c). The linear regression model explained a small amount of variance and was not statistically significant, *F*(1, 23) = 0.13, *p* = 0.72, beta = 7.46e − 03, 95% CI [− 0.04, 0.05], *R*^2^ = 0.01, BF_10_ = 0.39.

### Reach and lift kinematics

We conducted a series of paired *t* tests to examine whether the reach and lift kinematics differed between real and virtual conditions.

#### Maximum reach velocity

**Fig. 5:**
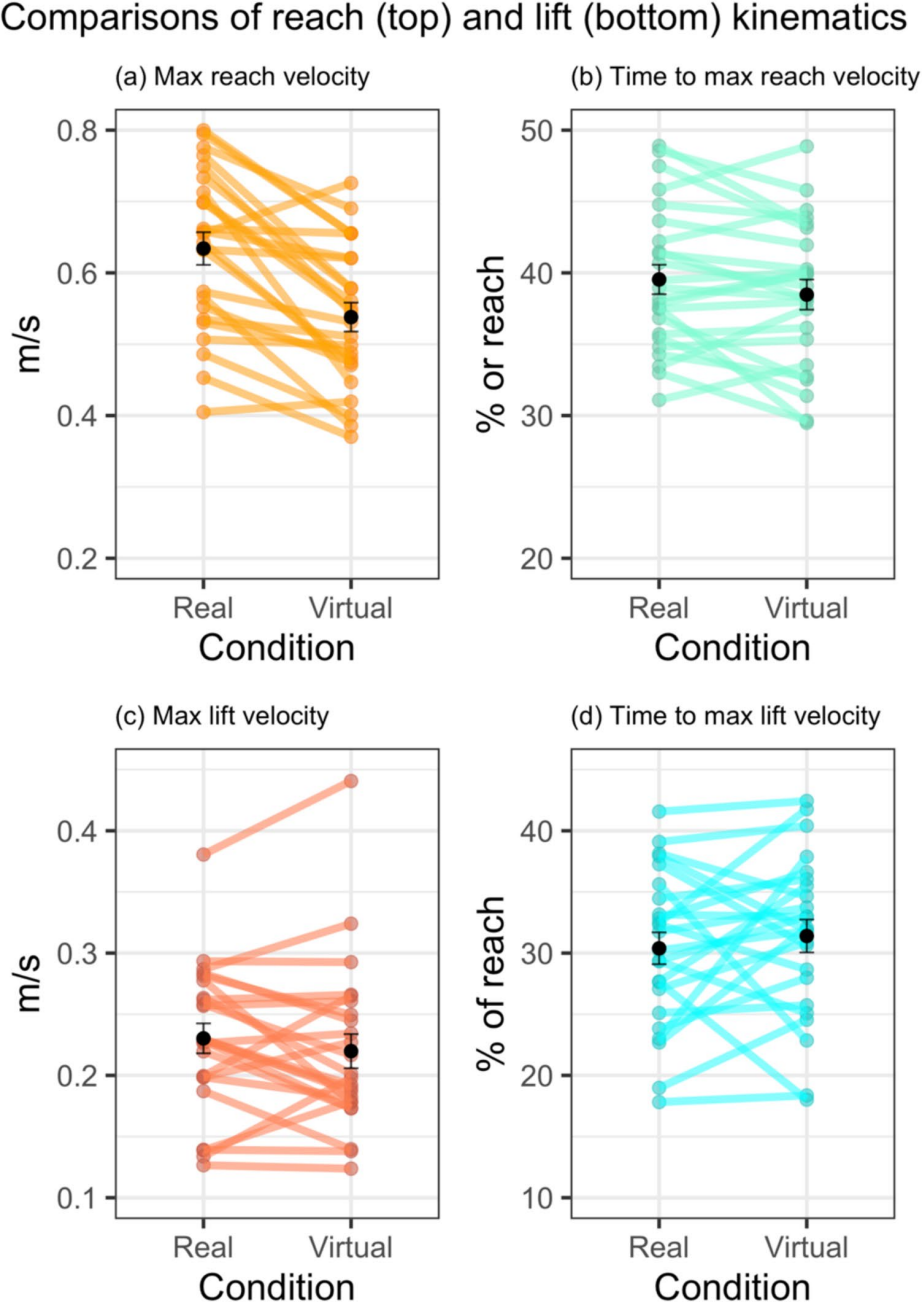
Between condition comparisons of reach and lift kinematics. *Note.* Panels **(a)–(d)** show raw data points in colours, with overlaid mean and standard error in black. (Colour figure online)

A paired *t* test indicated a statistically significant and large, mean difference = 0.10; 95% CI [0.06, 0.13], difference in maximum reach velocity between real and virtual conditions, *t*(23) = 5.74, *p* < 0.001, *d*_z_ = 1.20, corresponding to higher velocity reaches in the real condition. A Bayesian analysis indicated extreme evidence for the alternative hypothesis (BF_10_ = 2813.58; see Fig. 5a).

#### Time to maximum reach velocity

A paired *t* test indicated the difference in time to maximum reach velocity between real and virtual conditions, mean difference = 1.06, 95% CI [− 0.20, 2.32], was small and not statistically significant, *t*(23) = 1.75, *p* = 0.09, *d*_z_ = 0.36. A Bayesian analysis indicated anecdotal evidence in favour of the null (BF_10_ = 0.80; see Fig. 5b).

#### Maximum lift velocity

A Shapiro–Wilk test indicated that maximum lift velocity data deviated substantially from normality, so a Wilcoxon signed-rank test was used. This test indicated that there was not a statistically significant difference between the real and virtual conditions (*V* = 187, *p* = 0.30). A Bayesian analysis indicated anecdotal evidence in favour of the null (BF_10_ = 0.40; see Fig. 5c).

#### Time to maximum lift velocity

A paired *t* test for time to maximum lift velocity indicated that the difference between real and virtual conditions, mean difference =  − 1.01, 95% CI [− 3.67, 1.66], was small and not statistically significant, *t*(23) =  − 0.78, *p* = 0.44; *d*_z_ = − 0.16. A Bayesian analysis indicated anecdotal evidence in favour of the null (BF_10_ = 0.40; see Fig. 5d).

## Discussion

In this study, we compared the size–weight illusion and object lifting kinematics between real and virtual conditions. We explored whether the relative impact of prior expectations on perception may be shifted in VR, due to additional uncertainty associated with either expectations about the physical properties of virtual objects (leading to diminished influence of prior) or the perceptual information gleaned from the environment (leading to increased influence of prior). Given that the virtual environment we used was highly realistic, and visual size cues and physical objects were exactly matched between real and virtual conditions, we hypothesised that the novelty of the virtual conditions (e.g., see Yarossi et al., 2021) would induce a diminished influence of prior expectations, inducing a smaller SWI.

We observed that the normal of pattern of heaviness perceptions seen in previous SWI studies was present in the virtual condition, as there were large main effects of both object weight and size (see Fig. 2), which were comparable with physical reality. There were no interactions of presentation condition (real vs. virtual) with size or weight, suggesting that the virtual condition did not alter the experience of real or illusory mass. Turning to our primary research question, there was no conclusive evidence that presentation condition influenced the magnitude of the SWI. Despite a small- to moderate-sized reduction in the SWI in the virtual condition (*d* = 0.42), this effect did not reach statistical significance (*p* = 0.052), with Bayesian analysis suggesting only anecdotal evidence (BF_10_ = 1.25) for a between-condition difference. These findings contrast with those of Heineken and Schulte (2007), who reported a large reduction in the SWI in VR compared with the physical environment. This disparity could be a function of the increased realism of the VR environment and higher fidelity VR technologies used in the current study, which could have diminished the effects of the virtual presentation conditions. Alternatively, participants in the study by Heineken and Schulte did not lift the objects manually, but with a stick that ‘hooked’ the object. Future studies wishing to disentangle these issues should directly contrast lifting style with condition and could strengthen these conclusions further by parametrically varying aspects of the virtual environment’s fidelity.

It is worth noting how a change in the SWI in VR could result from a shift in either the *central tendency* or *the precision* of priors in VR. If beliefs can indeed be characterised as probability distributions, the posterior could be shifted by the mode of the prior being different in VR, or by the mode being the same, but more or less precise (i.e., a wider, flatter belief distribution). We recorded verbal estimates of prelift expectations about object weight to check whether they were indeed similar in VR (see Supplementary File 1 on OSF), which confirmed that people held very similar starting beliefs about the weight of the objects in both real and virtual conditions. As a result, any diminished effect of priors in VR in this task is likely due to a difference in precision of belief rather than central tendency. In future work, recording verbal estimates of confidence, which have been shown to accurately track belief precision (Heilbron & Meyniel, 2019), would be instructive in confirming this assumption.

Our hypothesis that lifting kinematics would be slower and more controlled in the virtual conditions was partly supported, with differences in reach but not lift kinematics. Reach velocities were clearly slower in the virtual condition indicating a more controlled approach to the object. One possibility is that uncertainty around depth perception in VR (Ping et al., 2020; Vienne et al., 2020) could have influenced reach behaviour. Alternatively, the absence of a realistic visible hand may have also affected the precision of open-loop movement control (Connolly & Goodale, 1999), although participants did execute their reaches confidently after a few practice trials in VR and did not have difficulty in grasping the object. That the time to maximum reach velocity did not differ between conditions suggests that the overall profile of the reach (i.e., acceleration at the start and deceleration at the end) did not substantively change, just the overall speed of the movement. Previous work has also shown that the lack of differences in lifting kinematics suggests that once the object had been grasped, participants lifted the objects in a similar fashion across the two conditions.

We hypothesised that higher presence scores would correlate with larger SWI scores because expectations would be stronger when participants felt more immersed in the virtual environment. Linear regression analyses indicated that there was a statistically significant relationship in the opposite direction, such that SWI scores were larger for participants with lower presence scores. A possible explanation for this surprising finding is that individuals who experienced a reduced sense of presence in VR (i.e., less accepting of the virtual environment as their reality) were relying almost entirely on predictive models from the real world (precise beliefs built up from repeated real-world object interactions). Consequently, they experienced a stronger illusion. By contrast, those that were highly immersed in the virtual world may have used new priors tied to the virtual context. As discussed previously, it may be that these expectations were less precise and therefore had a diminished impact on perception. Alternatively, individuals who felt particularly present in the scene could have been distracted from reporting their (in this case illusory) experience of object mass and/or failed to appropriately attend to the size of the object.

Future research should therefore investigate environments with varying levels of realism and fidelity to determine how visual similarity between real and virtual settings influences our expectations about virtual objects. An important question to explore is the extent to which participants transfer their learning about objects between virtual and real environments. This question of skill transfer is critical for the applications of VR in fields such as sports performance enhancement and rehabilitation (Bossard et al., 2008; Juliano & Liew, 2020; Yarossi et al., 2021). Given that expectations about the positive correlation between object volume and object weight can be overwritten with sufficient experience (Flanagan et al., 2008), it is essential to examine whether priors derived from within virtual environments affect real-world interactions.

An important caveat to the present findings is the role of individual differences in perceiving the uncertainty or novelty of virtual environments. It is conceivable that some individuals downweighted their learned priors about size and weight in VR, while others did the opposite, leading to an overall null effect. The aim of the present work was, however, to examine whether there was a general biasing effect of VR on perception, for which we found no strong evidence. Therefore, it is possible that more subtle individual differences in the size or direction of effect could be present but this was not the current focus. Additionally, we collected presence questionnaire data as an attempt to capture part of these individual differences in perception of the VR, with our results suggesting that perceived realism did not influence the difference in SWI between the conditions. Nonetheless, future work may wish to more closely examine individual difference factors, such as previous experience with VR or gaming, intolerance of uncertainty, visual and sensorimotor sensitivities, or susceptibility to motion/cyber sickness.

## Conclusions

Understanding the potential differences in perception and action between real and virtual environments is imperative for effectively harnessing immersive technologies for motor skill training, such as applications in sports performance enhancement and rehabilitation. If our interactions and predictive models in XR substantially deviate from those in the physical world, it may hinder the seamless transfer of knowledge and skills between XR and the real world, potentially giving rise to unintended effects in our physical surroundings. Numerous studies have investigated how human movements differ between physical reality and virtual environments, particularly focusing on how altered sensory inputs can change movement patterns. However, there has been a complete lack of research on how prior expectations in virtual environments might differ from those in the real world and how these expectations could impact our interactions. In this study, we demonstrated that the size–weight illusion, which is thought to be driven by prior expectations about the physical properties of objects, was present in virtual reality, and found no compelling evidence that it was reduced compared with the physical environment. Additionally, we corroborated previous findings that movements in virtual environments tend to be slower and more controlled, providing support for the notion that perception and action may be affected in different ways by immersion in a virtual environment (Harris et al., 2019).

## Data Availability

All relevant data are available online (https://osf.io/8gty7/).

## Footnotes

1 We also ran this analysis using the alternative method for computing the SWI magnitude by subtracting the average *z* score for the large objects from the average z-score for the small objects. The results are described in Supplementary File 2 on the OSF page.

## Code availability

All relevant code is available online (https://osf.io/8gty7/).

## References

[CR1] Alaker, M., Wynn, G. R., & Arulampalam, T. (2016). Virtual reality training in laparoscopic surgery: A systematic review & meta-analysis. *International Journal of Surgery,**29*, 85–94. 10.1016/j.ijsu.2016.03.034 26992652 10.1016/j.ijsu.2016.03.034

[CR2] Allen, K., Harris, D., Arthur, T., Wood, G., & Buckingham, G. (2023). Investigating how prior knowledge influences perception and action in developmental coordination disorder. *Quarterly Journal of Experimental Psychology,**77*(10), 2065–2075. 10.1177/17470218231214479 10.1177/17470218231214479 PMC11487901 37926854

[CR3] Arthur, T., Vine, S., Brosnan, M., & Buckingham, G. (2020). Predictive sensorimotor control in autism. *Brain,**143*(10), 3151–3163. 10.1093/brain/awaa243 32974646 10.1093/brain/awaa243

[CR4] Beck, L., Wolter, M., Mungard, N. F., Vohn, R., Staedtgen, M., Kuhlen, T., & Sturm, W. (2010). Evaluation of spatial processing in virtual reality using functional magnetic resonance imaging (fMRI). *Cyberpsychology, Behavior, and Social Networking,**13*(2), 211–215. 10.1089/cyber.2008.0343 20528281 10.1089/cyber.2008.0343

[CR5] Behrens, T. E. J., Woolrich, M. W., Walton, M. E., & Rushworth, M. F. S. (2007). Learning the value of information in an uncertain world. *Nature Neuroscience,**10*(9), 9. 10.1038/nn1954 17676057 10.1038/nn1954

[CR6] Bossard, C., Kermarrec, G., Buche, C., & Tisseau, J. (2008). Transfer of learning in virtual environments: A new challenge? *Virtual Reality,**12*(3), 151–161. 10.1007/s10055-008-0093-y

[CR7] Brock, K., Vine, S. J., Ross, J. M., Trevarthen, M., & Harris, D. J. (2023). Movement kinematic and postural control differences when performing a visuomotor skill in real and virtual environments. *Experimental Brain Research,**241*, 1797–1810. 10.1007/s00221-023-06639-0 37222777 10.1007/s00221-023-06639-0 PMC10348942

[CR8] Buckingham, G. (2014). Getting a grip on heaviness perception: A review of weight illusions and their probable causes. *Experimental Brain Research,**232*(6), 1623–1629. 10.1007/s00221-014-3926-9 24691760 10.1007/s00221-014-3926-9

[CR9] Buckingham, G. (2019). Examining the size–weight illusion with visuo-haptic conflict in immersive virtual reality. *Quarterly Journal of Experimental Psychology,**72*(9), 2168–2175. 10.1177/1747021819835808 10.1177/1747021819835808 30789088

[CR10] Buckingham, G., Ranger, N. S., & Goodale, M. A. (2011). The material–weight illusion induced by expectations alone. *Attention, Perception, & Psychophysics,**73*(1), 36–41. 10.3758/s13414-010-0007-4 10.3758/s13414-010-0007-4 21258907

[CR11] Charpentier, A. (1891). Analyse experimentale: De quelques elements de la sensation de poids [Experimental analysis: Some elements of the sensation of weight]. *Archives De Phisiologie Normale Et Pathologique,**3*, 122–135.

[CR12] Clark, A. (2013). Whatever next? Predictive brains, situated agents, and the future of cognitive science. *Behavioral and Brain Sciences,**36*(3), 181–204. 10.1017/S0140525X12000477 23663408 10.1017/S0140525X12000477

[CR13] Connolly, J. D., & Goodale, M. A. (1999). The role of visual feedback of hand position in the control of manual prehension. *Experimental Brain Research,**125*(3), 281–286. 10.1007/s002210050684 10229019 10.1007/s002210050684

[CR14] de Lange, F. P., Heilbron, M., & Kok, P. (2018). How do expectations shape perception? *Trends in Cognitive Sciences,**22*(9), 764–779. 10.1016/j.tics.2018.06.002 30122170 10.1016/j.tics.2018.06.002

[CR15] Eadie, A. S., Gray, L. S., Carlin, P., & Mon-Williams, M. (2000). Modelling adaptation effects in vergence and accommodation after exposure to a simulated virtual reality stimulus. *Ophthalmic and Physiological Optics,**20*(3), 242–251. 10897346

[CR16] Ellis, R. R., & Lederman, S. J. (1999). The material-weight illusion revisited. *Perception & Psychophysics,**61*(8), 1564–1576. 10.3758/BF03213118 10598470 10.3758/bf03213118

[CR17] Faul, F., Erdfelder, E., Lang, A.-G., & Buchner, A. (2007). G*Power 3: A flexible statistical power analysis program for the social, behavioral, and biomedical sciences. *Behavior Research Methods,**39*(2), 175–191. 10.3758/BF03193146 17695343 10.3758/bf03193146

[CR18] Flanagan, J. R., & Beltzner, M. A. (2000). Independence of perceptual and sensorimotor predictions in the size–weight illusion. *Nature Neuroscience,**3*(7), 737–741. 10.1038/76701 10862708 10.1038/76701

[CR19] Flanagan, J. R., Bittner, J. P., & Johansson, R. S. (2008). Experience can change distinct size-weight priors engaged in lifting objects and judging their weights. *Current Biology,**18*(22), 1742–1747. 10.1016/j.cub.2008.09.042 19026545 10.1016/j.cub.2008.09.042

[CR20] Friston, K. (2005). A theory of cortical responses. *Philosophical Transactions of the Royal Society b: Biological Sciences,**360*(1456), 815–836. 10.1098/rstb.2005.1622 10.1098/rstb.2005.1622 PMC1569488 15937014

[CR21] Friston, K. (2010). The free-energy principle: A unified brain theory? *Nature Reviews Neuroscience,**11*(2), 127–138. 10.1038/nrn2787 20068583 10.1038/nrn2787

[CR22] Giesel, M., Nowakowska, A., Harris, J. M., & Hesse, C. (2020). Perceptual uncertainty and action consequences independently affect hand movements in a virtual environment. *Scientific Reports*, *10*. 10.1038/s41598-020-78378-z 10.1038/s41598-020-78378-z PMC7749146 33339859

[CR23] Gregory, R. L. (1970). *The intelligent eye*. Weidenfeld and Nicolson.

[CR24] Harris, D. J., Arthur, T., de Burgh, T., Duxbury, M., Lockett-Kirk, R., McBarnett, W., & Vine, S. J. (2023). Assessing expertise using eye tracking in a virtual reality flight simulation. *The International Journal of Aerospace Psychology*, *0*(0), 1–21. 10.1080/24721840.2023.2195428

[CR25] Harris, D. J., Buckingham, G., Wilson, M. R., Brookes, J., Mushtaq, F., Mon-Williams, M., & Vine, S. J. (2020). The effect of a virtual reality environment on gaze behaviour and motor skill learning. *Psychology of Sport and Exercise,**50*, 101721. 10.1016/j.psychsport.2020.101721

[CR26] Harris, D. J., Buckingham, G., Wilson, M. R., & Vine, S. J. (2019). Virtually the same? How impaired sensory information in virtual reality may disrupt vision for action. *Experimental Brain Research,**237*(11), 2761–2766. 10.1007/s00221-019-05642-8 31485708 10.1007/s00221-019-05642-8 PMC6794235

[CR27] Hassan, E. K., Sedda, A., Buckingham, G., & McIntosh, R. D. (2020). The size-weight illusion in visual form agnosic patient DF. *Neurocase,**26*(5), 277–284. 10.1080/13554794.2020.1800748 32804579 10.1080/13554794.2020.1800748

[CR28] Heilbron, M., & Meyniel, F. (2019). Confidence resets reveal hierarchical adaptive learning in humans. *PLOS Computational Biology,**15*(4), e1006972. 10.1371/journal.pcbi.1006972 30964861 10.1371/journal.pcbi.1006972 PMC6474633

[CR29] Heineken, E., & Schulte, F. P. (2007). Seeing size and feeling weight: The size–weight illusion in natural and virtual reality. *Human Factors,**49*(1), 136–144. 10.1518/001872007779598028 17315850 10.1518/001872007779598028

[CR30] Helmholtz, H. V. (1860). *Treatise on physiological optics*. Dover.

[CR31] Hill, H., & Johnston, A. (2007). The hollow-face illusion: Object-specific knowledge, general assumptions or properties of the stimulus? *Perception,**36*(2), 199–223. 10.1068/p5523 17402664 10.1068/p5523

[CR32] Juliano, J. M., & Liew, S.-L. (2020). Transfer of motor skill between virtual reality viewed using a head-mounted display and conventional screen environments. *Journal of NeuroEngineering and Rehabilitation,**17*(1), 48. 10.1186/s12984-020-00678-2 32276664 10.1186/s12984-020-00678-2 PMC7149857

[CR33] Knill, D. C., & Pouget, A. (2004). The Bayesian brain: The role of uncertainty in neural coding and computation. *Trends in Neurosciences,**27*(12), 712–719. 10.1016/j.tins.2004.10.007 15541511 10.1016/j.tins.2004.10.007

[CR34] Kober, S. E., Settgast, V., Brunnhofer, M., Augsdörfer, U., & Wood, G. (2022). Move your virtual body: Differences and similarities in brain activation patterns during hand movements in real world and virtual reality. *Virtual Reality,**26*(2), 501–511. 10.1007/s10055-021-00588-1

[CR35] Körding, K. P., & Wolpert, D. M. (2004). Bayesian integration in sensorimotor learning. *Nature,**427*(6971), 244–247. 10.1038/nature02169 14724638 10.1038/nature02169

[CR36] Magdalon, E. C., Michaelsen, S. M., Quevedo, A. A., & Levin, M. F. (2011). Comparison of grasping movements made by healthy subjects in a 3-dimensional immersive virtual versus physical environment. *Acta Psychologica,**138*(1), 126–134. 10.1016/j.actpsy.2011.05.015 21684505 10.1016/j.actpsy.2011.05.015

[CR37] Mangalam, M., Yarossi, M., Furmanek, M. P., Krakauer, J. W., & Tunik, E. (2023). Investigating and acquiring motor expertise using virtual reality. *Journal of Neurophysiology,**129*(6), 1482–1491. 10.1152/jn.00088.2023 37194954 10.1152/jn.00088.2023 PMC10281781

[CR38] Mangalam, M., Yarossi, M., Furmanek, M. P., & Tunik, E. (2021). Control of aperture closure during reach-to-grasp movements in immersive haptic-free virtual reality. *Experimental Brain Research,**239*(5), 1651–1665. 10.1007/s00221-021-06079-8 33774688 10.1007/s00221-021-06079-8 PMC9701584

[CR39] McGurk, H., & MacDonald, J. (1976). Hearing lips and seeing voices. *Nature,**264*(5588), 5588. 10.1038/264746a0 10.1038/264746a0 1012311

[CR40] Merker, S., Pastel, S., Bürger, D., Schwadtke, A., & Witte, K. (2023). Measurement accuracy of the HTC VIVE Tracker 3.0 compared to Vicon System for generating valid positional feedback in virtual reality. *Sensors,**23*(17), 17. 10.3390/s23177371 10.3390/s23177371 PMC10490571 37687827

[CR41] Milgram, P., & Kishino, F. (1994). A taxonomy of mixed reality visual display. *IEICE Transactions on Information and Systems, D,**77*(12), 1321–1329.

[CR42] Morey, R. D., & Rouder, J. N. (2015). *BayesFactor: Computation of Bayes factors for common designs* (R Package Version 0.9.12–2) [Computer software]. https://CRAN.R-Project.Org/package=BayesFactor

[CR43] Naylor, C. E., Proulx, M. J., & Buckingham, G. (2022). Using immersive virtual reality to examine how visual and tactile cues drive the material-weight illusion. *Attention, Perception, & Psychophysics,**84*(2), 509–518. 10.3758/s13414-021-02414-x 10.3758/s13414-021-02414-x PMC8641965 34862589

[CR44] Neumann, D. L., Moffitt, R. L., Thomas, P. R., Loveday, K., Watling, D. P., Lombard, C. L., & Tremeer, M. A. (2018). A systematic review of the application of interactive virtual reality to sport. *Virtual Reality,**22*(3), 183–198. 10.1007/s10055-017-0320-5

[CR45] Niehorster, D. C., Li, L., & Lappe, M. (2017). The accuracy and precision of position and orientation tracking in the HTC Vive Virtual Reality System for Scientific Research: *I-Perception*, *8*(3). 10.1177/2041669517708205 10.1177/2041669517708205 PMC5439658 28567271

[CR46] Pek, J., Wong, O., & Wong, A. C. M. (2018). How to address non-normality: A taxonomy of approaches, reviewed, and illustrated. *Frontiers in Psychology*, *9*. https://www.frontiersin.org/articles/10.3389/fpsyg.2018.02104 10.3389/fpsyg.2018.02104 PMC6232275 30459683

[CR47] Peterson, M. A. (1999). Organization, segregation and object recognition. *Intellectica,**28*, 37–51.

[CR48] Peterson, M. A., & Gibson, B. S. (1994). Must figure-ground organization precede object recognition? An Assumption in Peril. *Psychological Science,**5*(5), 253–259. 10.1111/j.1467-9280.1994.tb00622.x

[CR49] Ping, J., Thomas, B. H., Baumeister, J., Guo, J., Weng, D., & Liu, Y. (2020). Effects of shading model and opacity on depth perception in optical see-through augmented reality. *Journal of the Society for Information Display,**28*(11), 892–904. 10.1002/jsid.947

[CR50] R Core Team. (2017). *R: A language and environment for statistical computing* [Computer software]*.* R Foundation for Statistical Computing. https://www.R-project.org/

[CR51] Rohrbach, N., Hermsdörfer, J., Huber, L.-M., Thierfelder, A., & Buckingham, G. (2021). Fooling the size–weight illusion—Using augmented reality to eliminate the effect of size on perceptions of heaviness and sensorimotor prediction. *Virtual Reality,**25*(4), 1061–1070. 10.1007/s10055-021-00508-3

[CR52] Rzepka, A. M., Hussey, K. J., Maltz, M. V., Babin, K., Wilcox, L. M., & Culham, J. C. (2022). Familiar size affects perception differently in virtual reality and the real world. *Philosophical Transactions of the Royal Society B: Biological Sciences*, *378*(1869). 10.1098/rstb.2021.0464 10.1098/rstb.2021.0464 PMC9745877 36511414

[CR53] Saccone, E. J., Landry, O., & Chouinard, P. A. (2019). A meta-analysis of the size–weight and material-weight illusions. *Psychonomic Bulletin & Review,**26*(4), 1195–1212. 10.3758/s13423-019-01604-x 31044361 10.3758/s13423-019-01604-x

[CR54] Seth, A. K. (2015). The cybernetic Bayesian brain: From interoceptive inference to sensorimotor contingencies. *Open MIND*. 10.15502/9783958570108

[CR55] Signorell, A., Aho, K., Alfons, A., Anderegg, N., Aragon, T., Arppe, A., . . . Borchers, H. W. (2019). *DescTools: Tools for descriptive statistics* (R Package Version 0.99, 28, 17) [Computer software]. 10.32614/CRAN.package.DescTools

[CR56] Slater, M. (2018). Immersion and the illusion of presence in virtual reality. *British Journal of Psychology,**109*(3), 431–433. 10.1111/bjop.12305 29781508 10.1111/bjop.12305

[CR57] Slater, M., Steed, A., McCarthy, J., & Maringelli, F. (1998). The influence of body movement on subjective presence in virtual environments. *Human Factors,**40*(3), 469–477. 10.1518/001872098779591368 9849105 10.1518/001872098779591368

[CR58] Slater, M., & Wilbur, S. (1997). A Framework for Immersive Virtual Environments (FIVE): Speculations on the role of presence in virtual environments. *Presence: Teleoperators and Virtual Environments*, *6*(6), 603–616. 10.1162/pres.1997.6.6.603

[CR59] Snow, J. C., & Culham, J. C. (2021). The treachery of images: How realism influences brain and behavior. *Trends in Cognitive Sciences*10.1016/j.tics.2021.02.008 10.1016/j.tics.2021.02.008 PMC10149139 33775583

[CR60] Trapp, S., & Bar, M. (2015). Prediction, context, and competition in visual recognition. *Annals of the New York Academy of Sciences,**1339*(1), 190–198. 10.1111/nyas.12680 25728836 10.1111/nyas.12680

[CR61] Usoh, M., Arthur, K., Whitton, M. C., Bastos, R., Steed, A., Slater, M., & Brooks, F. P. (1999). Walking > walking-in-place > flying, in virtual environments. *Proceedings of the 26th Annual Conference on Computer Graphics and Interactive Techniques—SIGGRAPH ‘99*, 359–364. 10.1145/311535.311589

[CR62] van Polanen, V., & Davare, M. (2019). Dynamic size-weight changes after object lifting reduce the size-weight illusion. *Scientific Reports*, *9*(1), Article 15697. 10.1038/s41598-019-52102-y 10.1038/s41598-019-52102-y PMC6821833 31666612

[CR63] Vienne, C., Masfrand, S., Bourdin, C., & Vercher, J.-L. (2020). Depth perception in virtual reality systems: Effect of screen distance, environment richness and display factors. *IEEE Access*, *8*, 29099–29110. IEEE Access. 10.1109/ACCESS.2020.2972122

[CR64] Wann, J. P., Rushton, S., & Mon-Williams, M. (1995). Natural problems for stereoscopic depth perception in virtual environments. *Vision Research,**35*(19), 2731–2736. 7483313 10.1016/0042-6989(95)00018-u

[CR65] Weir, P. L., MacKenzie, C. L., Marteniuk, R. G., Cargoe, S. L., & Frazer, M. B. (1991). The effects of object weight on the kinematics of prehension. *Journal of Motor Behavior,**23*(3), 192–204. 10.1080/00222895.1991.10118362 14766516 10.1080/00222895.1991.10118362

[CR66] Wolpert, D. M. (2007). Probabilistic models in human sensorimotor control. *Human Movement Science,**26*(4), 511–524. 10.1016/j.humov.2007.05.005 17628731 10.1016/j.humov.2007.05.005 PMC2637437

[CR67] Yarossi, M., Mangalam, M., Naufel, S., & Tunik, E. (2021). Virtual reality as a context for adaptation. *Frontiers in Virtual Reality,**2*, 139. 10.3389/frvir.2021.733076

[CR68] Yon, D., & Frith, C. D. (2021). Precision and the Bayesian brain. *Current Biology,**31*(17), R1026–R1032. 10.1016/j.cub.2021.07.044 34520708 10.1016/j.cub.2021.07.044

[CR69] Yu, A. J., & Dayan, P. (2005). Uncertainty, neuromodulation, and attention. *Neuron,**46*(4), 681–692. 10.1016/j.neuron.2005.04.026 15944135 10.1016/j.neuron.2005.04.026

[CR70] Zwislocki, J. J., & Goodman, D. A. (1980). Absolute scaling of sensory magnitudes: A validation. *Perception & Psychophysics,**28*(1), 28–38. 10.3758/BF03204312 7413407 10.3758/bf03204312

